# ﻿A digital multi-access key for easy identification of large tree species of ebony wood in Madagascar

**DOI:** 10.3897/phytokeys.253.134319

**Published:** 2025-03-04

**Authors:** Hasina N. Rakouth, Sylvie Andriambololonera, Bakolimalala Rakouth, Peter B. Phillipson, Porter P. Lowry II, Nicholas Wilding

**Affiliations:** 1 Plant Biology and Ecology Department, University of Antananarivo, Antananarivo, Madagascar Missouri Botanical Garden, Madagascar Research and Conservation Program Antananarivo Madagascar; 2 Missouri Botanical Garden, Madagascar Research and Conservation Program, B.P. 3391, Antananarivo 101, Madagascar University of Antananarivo Antananarivo Madagascar; 3 Missouri Botanical Garden, Africa and Madagascar Department, 4344 Shaw Blvd., St. Louis, Missouri 63110, USA Missouri Botanical Garden, Africa and Madagascar Program St. Louis United States of America; 4 Institut de Systématique, Évolution et Biodiversité (ISYEB), Muséum National d'Histoire Naturelle, Centre National de la Recherche Scientifique, Sorbonne Université, École Pratique des Hautes Études-Paris Science et Lettres, Université des Antilles, C.P. 39, 57 rue Cuvier, 75005 Paris, France Université des Antilles Paris France; 5 UMR PVBMT- Pôle de Protection des Plantes Université de La Réunion, La Réunion, France UMR PVBMT- Pôle de Protection des Plantes Université de La Réunion La Réunion France

**Keywords:** CITES, *Diospyros*, ebony, Lucid 4, Madagascar, multi-access key, precious woods

## Abstract

In 2013, all populations of the precious wood genera *Dalbergia* (Fabaceae) and *Diospyros* (Ebenaceae) from Madagascar were placed on CITES Appendix II in an effort to combat unsustainable and illicit over-exploitation and illegal exportation for the international market. The accompanying Action Plan adopted by CITES identified several information and capacity gaps, which undermine the sustainable and equitable management of these valuable resources. These gaps include the lack of practical, reliable tools to identify species along the entire value chain, from standing trees to cut wood and finished products. To address this need, we developed simple, user-friendly, multi-access keys for the two genera in Madagascar using the Lucid application. This new tool provides highly accurate identification of standing and felled trees to assist actors in the forestry, regulatory, and natural resource management sectors, including customs officials and law enforcement authorities as well as conservationists and protected area managers. In this paper, we focus on the development of the *Diospyros* identification tool. This interactive, electronic key employs 109 informative characters, including morphological features, emphasizing vegetative structures such as bark, stems, and leaves that are present even in the absence of flowers and fruits, in conjunction with eco-geographic characters (bioclimate, elevation, and geography). The key is supplemented with photos, illustrations, and a comprehensive glossary, to deliver accurate identification of the 88 *Diospyros* species that are large enough to be potential sources of commercially valuable ebony wood (≥ 20 cm DBH and/or ≥ 20 m height). This is the first use of Lucid to develop an identification key for species in Madagascar, paving the way for its application to other taxa for which practical electronic field identification is needed.

## ﻿Introduction

Over the last two decades, members of the precious wood genera *Diospyros* (Ebenaceae) and *Dalbergia* (Fabaceae) have faced rapidly growing pressure and worsening threats due to unsustainable levels of illicit exploitation, primarily within legally protected areas. Most of the ebony and rosewood timber obtained from them has been harvested illegally and exported for the international market, primarily to China (Global Witness & Environmental Investigation Agency 2009, 2010; Schuurman and Lowry 2009; Wilmé et al. 2009; Randriamalala and Liu 2010; Ballet et al. 2011; Randriamalala et al. 2011; Waeber et al. 2015; Andriamanana 2019).

As part of Madagascar’s obligations under the Convention on Biological Diversity (CBD) signed in Rio de Janeiro in 1992 (Stone 1996) and the Convention on International Trade of Endangered Species (CITES 1978), the government is responsible for ensuring the sustainable management and protection of all its species and for guaranteeing that their utilization does not threaten their survival. Measures to achieve a permanent halt to illegal exploitation in order to establish policies for rational and sustainable management of precious woods as well as for the equitable sale of these valuable resources have recently been initiated (Innes 2010; Pepke et al. 2015). Furthermore, in 2013, all Malagasy species of both *Diospyros* (which produces ebony wood) and *Dalbergia* (the primary source of rosewood) were listed on CITES Appendix II in an attempt to reduce over-exploitation and commercial trafficking (Andriambololonera et al. 2013; CITES 2013a, b). That same year, at the 16^th^ CITES Conference of Parties, an Action Plan was adopted (Decision 16.152 and subsequently Decision 17.203) requiring the establishment of an embargo on the export of wood stockpiles that had accumulated following seizure by the Malagasy authorities and prohibiting commercial exchange of ebony and rosewood (Andriamanana 2019). Yet despite these efforts, exploitation has continued (Ballet et al. 2011; Mason et al. 2016; Ratsimbazafy et al. 2016). Moreover, significant gaps were identified regarding the scientific knowledge base of these precious wood genera, which prompted a series of recommendations in the Action Plan to address them (CITES 2018).

One of the most important issues identified in the Action Plan was the inability to provide correct scientific names and reliable identifications for species, which are the principal unit of CITES management and a key element of biodiversity. This situation is due in large part to the lack of efficient identification tools (Delaunay 2020). Moreover, actors involved in the Malagasy forestry sector use generalized designations for various types of precious wood, either in the form of common (vernacular) names (which vary regionally) or commercial names based on the color and other characteristics of the wood being exploited. However, these names often apply to two or more distinct species, and they are frequently used inconsistently. For example, all species of *Diospyros* with black heartwood are called ‘ebony’ or ‘hazomainty’ in Malagasy, and species of *Dalbergia* with red or deep pink-violet heartwood are called ‘rosewood’, or ‘andramena’. Consequently, commercial and scientific names represent fundamentally different concepts in the context of the sustainable management of these important resources and the species from which they are sourced (Rakotovao et al. 2012). Addressing the challenges associated with the accurate identification and naming of species is a major hurdle for the eventual relaxation or lifting of the current embargo on these two genera by means of establishing a non-detriment finding (NDF) for a species whose exploitation would be possible without compromising its survival (Mason et al. 2016) or by removing them for CITES Appendix II. Therefore, it is crucial to develop methods and tools that allow for the accurate, precise, and unambiguous identification of species. This is particularly important when evaluating and managing populations of precious wood species, as it informs decision-making regarding their conservation, management, and exploitation.

To help address the objectives of the CITES Action Plan for Madagascar, a consortium was established in 2017 to develop scientific knowledge and tools in support of the sustainable management of the country’s precious wood genera *Dalbergia* and *Diospyros* (the consortium is known by its French acronym G3D – Gestion Durable des bois précieux *Dalbergia* et *Diospyros* de Madagascar). The management and control of these species requires reliable identification along the entire value chain, from standing trees and cut logs in the forest to sawn wood and finished products, and each of these stages requires its own methods and tools. A multi-disciplinary approach was therefore adopted that comprises four complementary components:

- Taxonomy (documentation of populations, field collection of representative samples, and clarification of species delimitation).
- Development of practical, reliable identification tools for standing trees and for specimens with leaves, flowers and/or fruits.
- Establishment of forensic identification methods based on the study of a) comparative wood anatomy, b) DNA sequencing (barcoding), and c) near infrared spectroscopy.
- Development of effective management strategies for the conservation of wild populations.

Collectively the main objective of these components is to establish a solid scientific base of research material and associated knowledge to inform accurate delimitation and identification of potentially exploitable species of *Diospyros* and *Dalbergia* for their effective management and for forensics. As part of this initiative, work was undertaken to develop practical identification tools for both genera based on morphological and eco-geographic characters, as part of the G3D taxonomy component led by the Missouri Botanical Garden’s Madagascar Program.

Identification keys are generally based on morphological characters of plant organs and are primarily used to distinguish species based, as far as possible, on easily observed features. Keys primarily make use of the inflorescence structure and of characteristics of the flowers and fruits, often complemented by characters of the leaves as well as other aspects such as the plant’s growth form or habit. They are nearly always dichotomous and are generally structured for publication in scientific articles or in floras and guidebooks. In this type of key, the sequence in which information is presented to the user is pre-determined by the author, usually by offering the user two mutually exclusive choices (a “couplet”) at each step by means of a text description of one or more alternative diagnostic characters (Judd et al. 1999; Hagedorn et al. 2010; Griffing 2011), leading either to another couplet or to an identification. However, when attempting to make an identification in the field, and especially if confronted with a sterile individual (lacking flowers and fruits, which is often the case when conducting forest inventories), dichotomous keys based primarily or exclusively on reproductive organs are difficult if not impossible to use. Even when flowers and/or fruits are available, a key that employs specialized terminology can present problems that render it impractical to users not familiar with technical jargon. Moreover, published dichotomous keys cannot easily be modified or updated if new information becomes available or new, morphologically similar species are recognized (Hardisty and Roberts 2013; Mangold 2013; Zuquim et al. 2017).

One way to overcome these constraints is to develop keys that combine traditionally used characters with new, informative features that have been underutilized or ignored, such as those involving vegetative structures, which are more likely to be observable regardless of the phenological stage, even in the absence of flowers and fruits. Leaf features are particularly pertinent in that they often enable recognizing and distinguishing among closely related taxa (Hickey et al. 1999, Ellis et al. 2009). Similarly, while the utility of bark features for recognizing tree species is well known in temperate areas for genera such as *Acer*, *Betula*, *Picea*, *Populus*, and *Quercus*, among others (Biswas et al. 2016; Carpentier et al. 2018; Wu et al. 2021; Juola et al. 2022), bark characters have not been used widely in tropical regions and very little in Madagascar. Although bark characters have not been utilized previously for species delimitation and recognition of Malagasy *Diospyros*, field observations have clearly revealed significant variation among species, suggesting that they are of potential use for field identification. Likewise, eco-geographic characters such as bioclimate, vegetation type, and elevation, which have proven to exhibit species-specific patterns and to be highly informative for species delimitation (Lowry et al. 1999; Vences et al. 2009; Rabarimanarivo et al. 2015; Crameri et al. 2022), are also of potential value for informing accurate identification.

In the age of global access to information via the internet and the rapid development of bioinformatics tools and technologies, it is now possible to build interactive and richly illustrated, multi-access identification keys that are simple, practical, and efficient. Moreover, these keys can be accessed through portable platforms that function independently, without needing an internet connection. This is precisely the type of tool that is required to enable reliable identification of Malagasy *Diospyros* and *Dalbergia*, employing a data matrix (or character/species) multi-access type of key, a model that is now being widely used (Begum et al. 2012; Wati et al. 2018). Early multi-access keys were based on the punch card approach (Hansen and Rahn 1969), in which an array of characters with two or more states that are not necessarily mutually exclusive are presented to the user, who is not required to follow a pre-determined sequence of steps (in contrast to conventional keys), but which can instead be used to select features in the order that best corresponds to the material being identified. This type of key is particularly advantageous when dealing with incomplete material (Judd et al. 1999, 2002). Several computer programs have recently been developed to produce interactive, matrix-based, multi-access identification keys (Dallwitz 2007; Gaubert et al. 2008; Hagedorn et al. 2010) such as DELTA and INTKEY (Dallwitz 1993), FRIDA (Martellos 2010), LINNAEUS (Wati et al. 2018), Lucid (Norton et al. 2000), and Xper versions 2 et 3 (Ung et al. 2010; Vignes-Lebbe et al. 2016; Lombard et al. 2021). They all exploit practical and flexible methods for coding characters, and some can incorporate illustrations (photos, drawings, and maps), as well as videos, links to internet sites, and a glossary. The major advantages of these keys are that they facilitate identification even when material is incomplete and are accessible for a wide range of users, including those less familiar with the organisms being identified than professional taxonomists.

One of the main objectives of the taxonomic component of the G3D project is to produce a tool that can be used at the beginning of the precious wood value chain to obtain a reliable species identification for each potentially exploitable tree of *Diospyros* and *Dalbergia*, a required step for the sustainable management of these resources. To meet this need, the identification tool must work reliably for trees that are still standing or that were recently felled, even if flowers or fruits are absent. After a careful comparison of the advantages and drawbacks of each of the applications mentioned above, Lucid was considered to be the best adapted to meet Madagascar’s current needs for accurate field identification. Lucid has already proven to be effective for the development of practical identification keys for a wide diversity of objects, ranging from minerals to fossil bacteria and living animals, such as certain insect groups, as well as algae and vascular plants (many examples in these and other groups can be found on the Lucid website: http://lucidcentral.org/).

The work presented in this paper summarizes the development of a practical key using Lucid 4 to facilitate the accurate and reliable identification of Malagasy species of *Diospyros* that are potential sources of commercially valuable ebony wood. As for large tree species of *Dalbergia* (Phillipson et al. 2023), a separate key has been developed using the same software and following the same principles and methods but is not discussed further in this paper.

*Diospyros* comprises an estimated 903 species of which 763 have been described to date, distributed across the main tropical regions (see the following for examples of recent taxonomic accounts of *Diospyros* spp. from various parts of the world: Tang et al. 2019; Schatz et al. 2021b; Puglisi et al. 2022; Hassler 2023; POWO 2023) and the remainder are currently in the process of being formally described. Madagascar represents one of the main centers of diversity for the genus, as indicated by the first comprehensive revision for the island published by Perrier de la Bâthie (1952a, b), who recognized a total of 97 species. However, collections and field observations made over the following decades clearly revealed the presence of far greater species diversity, and this catalyzed a new effort to document and describe Malagasy *Diospyros* starting some 15 years ago. This has led to the recognition of an estimated 285 species on the island, 151 of which have now been described and all but two of which are endemic to Madagascar (Schatz and Lowry 2018; Schatz and Lowry 2020; Schatz et al. 2020, 2021a, 2021b; Linan et al. 2021; Rakouth et al. 2023; Linan et al. 2024; Mestre et al. 2024), while an additional ca. 140 endemic species remain to be described (Madagascar Catalogue 2024). Field work conducted as part of the G3D project has contributed significantly to the available collection base, whose taxonomic component has now clarified the delimitation and completed the description of all 88 ‘large tree’ species (CITES 2024; Lowry et al. 2024), each of which is regarded as a potential source of commercially valuable ebony wood by virtue of the fact that it has been documented to reach a diameter at breast height (DBH) of ≥ 20 cm and/or a height of ≥ 20 m (Schatz et al. 2021b).

Species of *Diospyros* are found throughout all regions of Madagascar, but depending on a given species’ ecological preferences (altitude, substrate, habitat, etc.), they will generally be found in just one or two of Madagascar’s principal native vegetation types, including evergreen humid forest in the east, woodland or shrubland in the west, and deciduous dry forest in the north (often on karstic limestone, known as ‘tsingy’) or the south, as well as spiny thickets in the south and southwest (species of *Diospyros* are rare in montane vegetation). Some have wide geographical ranges, occurring across a large proportion of one or two of the island’s main bioclimatic regions, as defined by Cornet (1974) and simplified by Schatz (2000), while others have highly restricted ranges (Madagascar Catalogue 2024). A species’ eco-geographic preferences comprise valuable information that can complement morphological characters for developing identification keys (Wati et al. 2018; Lombard et al. 2021). With this in mind, we tested the value of including eco-geography in building our identification tool for large tree *Diospyros* species using the Lucid platform.

The tool we present here was designed primarily to assist an array of actors involved in various aspects of the management and control of Madagascar’s forestry sector, in particular with regard to inventories of standing trees. These stakeholders include forestry agents, operators involved in wood harvest (including both landowners and concession holders), managers of protected areas and other conservation sites, customs officials, and officers of the judiciary police, among others. Moreover, because the key we have developed enables users to identify dried collections (i.e., herbarium specimens) as well as living material, it will also be of value to researchers, field botanists, and students to improve their knowledge of the Malagasy species that are potential sources of commercially valuable ebony wood and strengthen their ability to recognize them.

## ﻿Materials and methods

### ﻿Lucid 4 software

For an identification tool to perform well and respond to Madagascar’s needs for managing precious woods by enabling accurate identification of standing trees and dried specimens, it must be simple, practical, interactive, and portable. Among the various programs available to produce electronic, matrix-based or multi-access keys, we selected Lucid version 4.0.4 (https://www.lucidcentral.org), which meets all of Madagascar’s needs for identifying large tree species of *Diospyros* in the field and herbarium. To our knowledge, Lucid keys have never been used before in Madagascar for species-level identification, even though keys can be distributed free of charge; only the developers are required to obtain a license to produce an operational tool. Utilization of a Lucid-based key does not require internet access, and as a key it can easily be modified, corrected, and updated with little effort by the developer. However, the main reason we opted to employ this program is because it is simple to use and functions without an internet connection, making it particularly well suited for field identification in Madagascar, where there is usually no internet access. Moreover, Lucid keys can easily be shared and transferred (by e-mail, USB key, on-line download, etc.) and can be used freely in any browser and on a wide range of platforms (laptops and tablets, or smartphones via a mobile application). Lucid is also practical and intuitive, allowing users to access a wide range of visual and text-based descriptors as well as species profiles (Vignes-Lebbe et al. 2016; Pinel et al. 2017). The developer is provided with an efficient interface containing all the tools and functionality needed to organize and deliver supplementary information to the user (photos, line drawings, links to on-line sites, glossaries, etc.) for each species and for important or difficult-to-interpret characters, greatly increasing the user’s ability to grasp and understand terminology, thus improving their ability to obtain an accurate identification. To optimize the functionality and utility of the Lucid-based tool we have developed, it was designed to perform well even in the absence of flowers and fruits and to remain robust and reliable even if the user misinterprets some characters. The tool thus provides efficient, multi-access entry according to the user’s needs and can deliver rapid and accurate identifications as well as valuable diagnostic information.

### ﻿Taxa

Among the ca. 285 species of *Diospyros* recognized in Madagascar, 88 develop into large enough trees to be potentially exploitable for ebony wood (CITES 2024; Lowry et al. 2024) and are therefore included in the Lucid key we have developed. Each species has its own particular geographic and bioclimatic distribution range, reflecting its ecological preferences. For example, within the island’s humid bioclimatic zone, *D.squamosa* is widely distributed in dense, humid, evergreen forests of the northwest and along the eastern escarpment (Schatz and Lowry 2020), whereas *D.littoralis* is restricted to littoral forests on sand near the eastern coastline (Schatz et al. 2021a). Other species have narrow ranges and are known from only a few sites, such as *D.lowryi* and *D.ultima*, both from low elevation humid forests in the northeast and known from only 2 and 4 localities, respectively (Schatz and Lowry 2018; Schatz et al. 2021a; Madagascar Catalogue 2024). Some species of *Diospyros* occur in dry, semi-deciduous forest on karstic limestone in the extreme north of the island, such as *D.vescoi* (which has a rather large range) and *D.crassifolia* (much more narrowly distributed) (Linan et al. 2021). Still other species occur in areas with similar vegetation in the west, such as *D.tropophylla* and *D.sakalavarum*, which have fairly large ranges, and *D.subtrinervis*, known from a single locality. The risk of extinction for each of the 88 large tree species was assessed between 2018 and 2024 according to the IUCN Red List criteria (IUCN SSC 2012, 2024). A total of 45 species (51%) were assessed as threatened, including 5 that were found to be Critically Endangered (CR), 18 Endangered (EN), and 22 Vulnerable (VU), whereas 11 species were assessed as Near Threatened (NT), and 31 were regarded as Least Concern (LC). One species was Data Deficient (DD) (IUCN 2024).

### ﻿Building the key

The key was constructed in four steps, implemented in the application’s key-building interface (Lucid Builder), which comprises three tabs at the base of the tree panels, [Tree View], [Spreadsheet Scoring] and [Score Analyser], as follows:

1. Data compilation within the [Tree View] tab.
2. Data coding and entry within the [Spreadsheet Scoring] tab.
3. Development of the key.
4. Testing, feedback, and improvement.

The [Score Analyser] tab provides analyses of character differences and species polymorphism to assess and indicate where improvements can be made to the key.

### ﻿Data compilation within the [Tree View] tab

#### ﻿Taxonomic entities backbone

The first tab [Tree View] enables the developer to view the structure (sequence and hierarchy) of characters and character states in the ‘features’ panel as well as a list of the species in the ‘entities’ panel. The taxonomic entities backbone was established by alphabetically entering the names of the 88 large tree *Diospyros* species in the [TreeView] tab. The species for which the key was developed are listed in Appendix 1 and can also be found on a dedicated page via the Catalogue of the Plants of Madagascar (Lowry et al. 2024).

#### ﻿Features backbone

The initial step of selecting characters and scoring character states involved preparing a list of potentially informative features of the stems, leaves, flowers, and fruits that vary among the large tree species of Malagasy *Diospyros*. Additional characters that could facilitate reliable field identification were then incorporated, in particular those that can only be observed in fresh material, including macroscopic features of vegetative organs such as the bark, which are easily observable throughout the year, even when an individual tree is sterile. For a key based exclusively on morphological data, the assumption is usually that any given species can occur anywhere within the geographic area being considered. This is clearly not the case, however, for *Diospyros* in Madagascar. We therefore compiled additional information on the ecological preferences of each species (bioclimate, altitudinal range, and vegetation type) as well as the potential geographic range for each species, to improve ease of identification by reducing the list of candidate species for a given site (Wati et al. 2018). The final selection of character features and the corresponding terminology included those that were determined to be the most pertinent for discriminating among the species and the easiest for users to interpret. The list of characters and states was entered into the ‘features’ panel on the [TreeView] tab of Lucid Builder. The features backbone of the key for the 88 species comprises data for morphological and eco-geographic characters, including a total of 109 characters and 356 character states (Appendix 2).

#### ﻿Compilation of data on morphological characters

**Figure 1.**
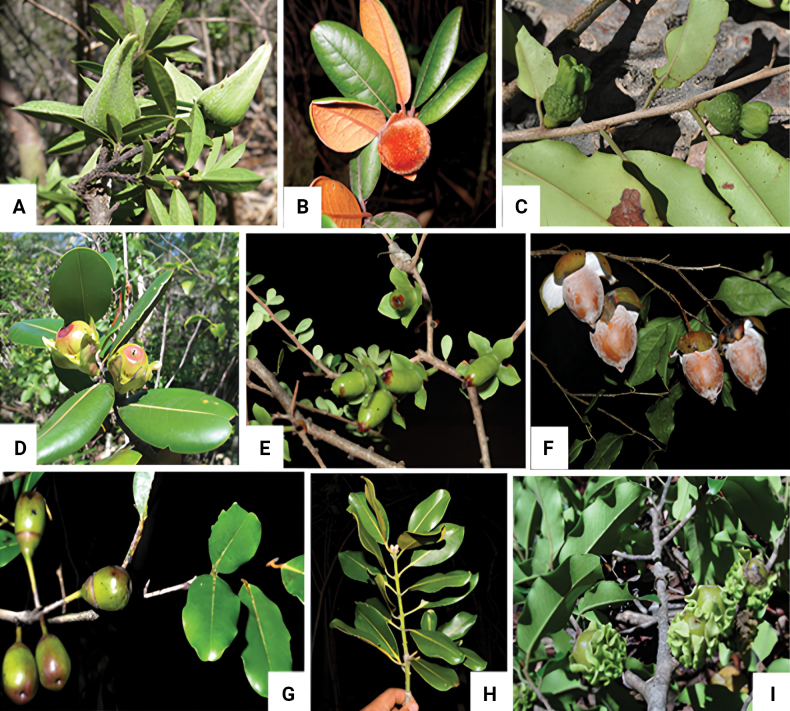
Photographs of selected large tree species of *Diospyros*, showing various morphological features of their leaves, fruit, and fruiting calyx **A***D.aculeata*, leaf apices terminating in a spine and fruiting calyx completely enclosing the fruit (photograph by F. Ratovoson) **B***D.antsirananae*, densely pubescent leaves and fruit, with reddish brown to ferruginous trichomes (photograph by S. Rakotonandrasana) **C***D.bernieriana*, margins of the leaves undulate and fruiting calyx expanding to enclose the fruit completely and form a prominent collar (photograph by G.E. Schatz) **D***D.crassifolia*, coriaceous, elliptic leaves and erect fruiting calyx lobes (photograph by S. Rakotonandrasana) **E***D.humbertiana*, small, obovate leaves and fruiting calyx with 4 or 5 spreading lobes (photograph by P. Lowry) **F***D.labatiana*, fruit surface and inner portion of calyx lobes covered by a white waxy substance (photograph by S. Andrianarivelo) **G***D.maculata*, glabrous leaves and fruit with an entire (unlobed), cupuliform calyx (photograph by P. Lowry) **H***D.parifolia*, subopposite to opposite, coriaceous, glabrous leaves (photograph by S. Andrianarivelo) **I***D.plicaticalyx*, lenticellate, gray/grayish twigs and fruiting calyx with undulate and plicate margins (photograph by G.E. Schatz).

Data on morphological character states exhibited by each species were primarily collected from descriptions available in the literature, complemented and refined based on examination of specimens available in the herbaria of the Parc Botanique et Zoologique de Tsimbazaza, Antananarivo, Madagascar (TAN), the Missouri Botanical Garden, St. Louis, Missouri, USA (MO), and the Muséum National d’Histoire Naturelle (MNHN), Paris, France (P), as well as scans of selected specimens available online. This was further enriched with field observations recorded by collectors on specimen labels (Lombard et al. 2021) and photos of living material, notably taken as part of the G3D project, showing details of habit, branching, bark, leafy branches, flowers, and fruits, which provide more precise information on character states seen on standing trees, whereas information obtained from the literature and pressed specimens is more informative for making identifications of herbarium material (Fig. 1, Appendix 2).

**Figure 2.**
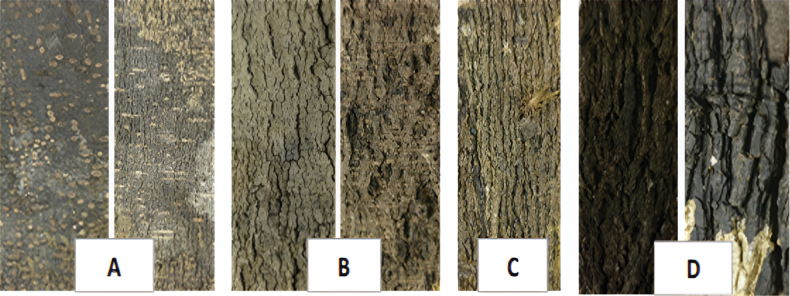
Examples of the three bark characters utilized in the Lucid key for Malagasy *Diospyros* (texture, presence of distinctive structures, and color) **A** smooth, lenticellate, gray/grayish bark of *D.ferrea***B** smooth, fissured, gray/grayish to light brown bark of *D.chitoniophora* (left) and *D.bardotiae* (right) **C** rugose, light brown bark with longitudinal striations of D. *brevipedicellata***D** rugose, deeply fissured, cracked, dark brown to black/blackish bark of *D.clusiifolia* (left) and *D.toxicaria* (right) (all photographs by H.N. Rakouth).

As mentioned above, bark characters have been shown to be of value for species identification in temperate genera but have been less widely used in the tropical areas, including Madagascar, where scientists have only rarely used bark features. In the absence of a standard method for describing bark characters, we drew from the works of several authors (Letouzey 1969; Junikka 1994; Rakotovao et al. 2012; Biswas et al. 2016; Carpentier et al. 2018) to identify those of potential value for Malagasy *Diospyros*. Three types of easy-to-observe characters were retained: bark surface texture (smooth, fissured, scaly, rugose), the presence of distinctive structures (lenticels, fissures, longitudinal and transverse striations, scales, a crust, crevasses, etc.), and overall color, both *in vivo* and *in sicco* (Fig. 2). To compile information on these characters for each species, we examined photos of tree trunks and bark samples taken in the field, along with high resolution images of dried bark material associated with herbarium specimens (Fig. 2). High resolution photos were taken at the Scientific Imaging Workshop (UAR 2700 2AD, BAOBAB facilities, DIM-MAP Île-de-France, CNRS and MNHN) located at the Muséum in Paris. For Madagascar, the use of bark features to identify species for the development of a practical identification tool represents pioneering work.

#### ﻿Compilation of data on eco-geographical characters

Information on ecological preferences and geographic distribution is also useful for facilitating the identification of Malagasy species of *Diospyros* given that each of them has its own distinctive specificities. We used QGIS (3.16.8 with GRASS 7.8.5) to visualize distribution data for each species (as a .kml points layer) and to record their eco-geographic characteristics using three key descriptors:

- Madagascar’s five main bioclimatic regions (humid, subhumid, dry, subarid, and montane), based on the bioclimatic map of Cornet (1974) as simplified by Schatz (2000).
- Five altitudinal classes between 0 and 2500 m (in 500 m increments); < 500 m, < 1000 m, < 1500 m, < 2000, and 2000 m or more.
- Potential geographic distribution: actual and potential presence in Madagascar was recorded in cells of 1°×1° resolution; potential presence was extrapolated from known occurrence points.

### ﻿Data scoring within the [Spreadsheet Scoring] tab

**Figure 3.**
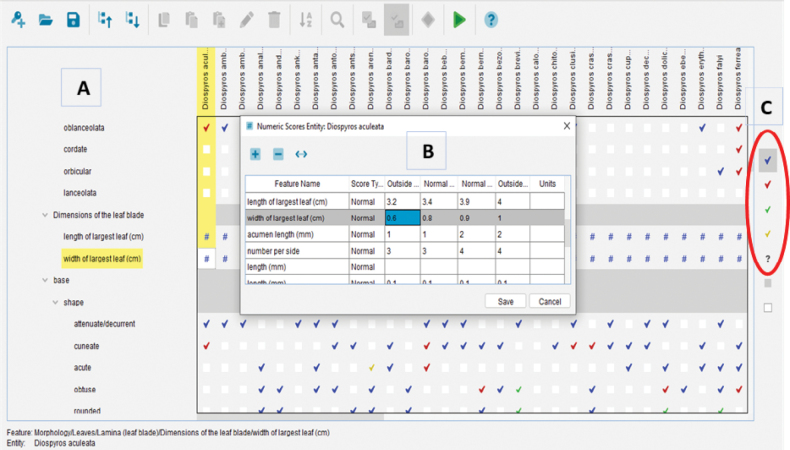
**A** screenshot of the *Diospyros* Lucid key-builder showing the data matrix table and the methods for scoring character data **B** inner pop-up table for numeric scores (#) of quantitative characters (here, width of largest leaf in cm). **C** The 5 types of usable interpretation scores for qualitative characters colored differently depending on the level of the character’s certainty: verified and unambiguous (blue), possible or likely to be misinterpreted (red), rare (green), rare and prone to misinterpretation (yellow), uncertain (?).

Scoring of features and states that correspond to each species was done for both qualitative and quantitative characters using the second tab in the key-building interface [Spreadsheet Scoring], which contains the data matrix of characters and species. Those that can potentially be misinterpreted, e.g. certain leaf shapes (elliptic, oblong, ovate, etc.), can be recorded in Lucid in a way that accommodates for potential user error. By using this feature, species are retained in the final list of potential results that would otherwise have been excluded due to the incorrect selection of a character state that is in fact not found in material belonging to the taxon. This is a helpful option since the interpretation of character states is not always straightforward and can vary between users. For a species that exhibits morphological variability, all possible character states are coded, enabling the user to select more than one state for a polymorphic feature. On the other hand, even if certain character states have not actually been observed on material of a given species, the person building the key can nevertheless select states that could reasonably be expected to be expressed and could therefore be encountered by a user or could be inadvertently selected due to a misinterpretation. To record information on the reliability of the states known for a particular character, a blue symbol is selected when the interpretation is verified and unambiguous, whereas a red symbol is chosen in situations where misinterpretation by the user is possible or likely. Rare or exceptional character states are indicated by a green symbol and those that are both rare and prone to misinterpretation are indicated in yellow. Finally, a question mark is used for characters whose state(s) is (are) uncertain or for which data are unavailable (Fig. 3). The Lucid tool for Malagasy *Diospyros* has thus been designed to function regardless of which plant organs are available for identification and to be reliable even when there is a risk of potential errors for certain characters due to user misunderstanding or misinterpretation.

Quantitative characters were counted or measured to code numerical values (#) in the data matrix table. This information was recorded in a table comprising four columns: outside minimum value, normal minimum value, normal maximum value, and outside maximum value, in which the normal values are calculated as the most frequent class of observations [normal min – normal max] in all measured samples. For example, in Fig. 3, the normal values are comprised in the class [0.8–0.9 cm] of the character “width of the largest leaf”, among 10 measured leaves. Counts or measurements were systematically made on an organ or structure regarded as being fully mature, and in order to standardize and facilitate comparison, the largest values were always used. For example, leaf dimensions were measured on the largest leaf on a leafy branch (Fig. 3).

**Figure 4.**
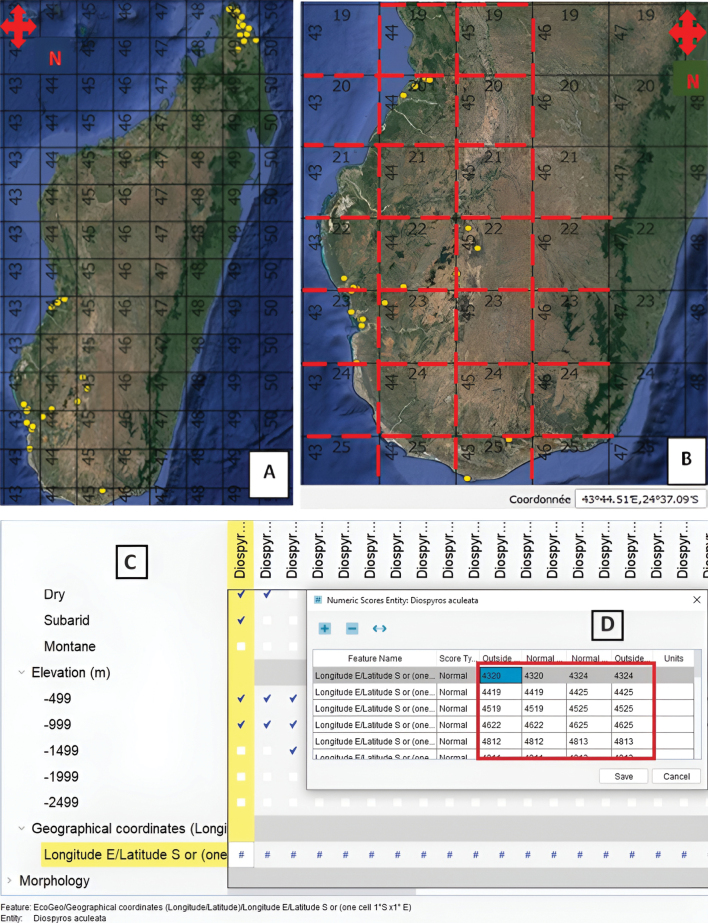
**A, B** screenshot from QGIS 3.16.8 **C, D** screenshot of Lucid key builder **A** current distribution map visualized on QGIS within Madagascar **B** map of the southern part of Madagascar showing grid cells (red dashes) encompassing the potential range visualized on QGIS **C** some of the eco-geographic features employed (elevation and geographic coordinates) **D** coordinate position coding system using the degree values of each 1 × 1-degree cell.

To score the potential geographic distribution of each species treated in the key, a grid comprising 75 cells, each with an area of 1°×1°, was superimposed over a map of Madagascar, which is situated between 11° to 25° S latitude and 43° to 50° E longitude. Taking into consideration the known range of each of the 88 *Diospyros* species as well as its eco-geographic preferences, the potential geographic range was scored in the appropriate cells. The convention used to name a given cell was based on the degree values for longitude and latitude corresponding to the points it encompasses. For example, the cell containing the coordinates of a population of *D.aculeata* located at 45°16'23"E longitude and 22°54'05"S latitude was labeled 4522, as shown in Fig. 4A–D. This allowed for scoring of longitudinal bands of presence, which, given the shape of Madagascar, were comparatively fewer in number than the alternative, using latitudinal bands of presence. Hence, the potential distribution of each species was characterized as a range within one or more longitudinal bands, and these were entered into Lucid as a quantitative variable with multiple independent ranges. We intentionally used a conservative approach for the process of estimating potential range, including all grid cells in which the species could reasonably be anticipated to occur. The resulting potential distribution was then systematically reviewed and validated by comparison with expert information provided by field botanists and taxonomists working on Malagasy *Diospyros*.

### ﻿Development of the key

The process of developing the *Diospyros* key involved making changes and fine-tuning it to enhance both its functionality and visual aspects in an effort to make it as user-friendly and straight forward as possible while retaining the ability to incorporate various types of supplementary information to improve identification accuracy. As English is the default language for Lucid, it was utilized in the development of the prototype *Diospyros* key. Considering the varying levels of botanical knowledge and familiarity with terminology among users in Madagascar, the key was designed to employ concise terminology for characters (features) while avoiding complex technical terms. Nevertheless, certain scientific terms were retained to maintain precision. For example, the technical term “brochidodromous” was used to define leaf venation in which secondary veins do not terminate at the margin but join to form a series of prominent arches, which form a sub marginal nerve (Ellis et al. 2009).

#### ﻿Media tool function

To augment the utility of the key, images were incorporated for each species, including photos of living plants as well as details of particular organs and distinctive features, accompanied by scans of herbarium specimens and line drawings as exemplars. Using the image viewer function of Lucid, the user can thus scroll among images and zoom in as needed to facilitate comparison with the material being identified. Selected images were recomposed to highlight key characters and character states in the ‘features’ window, and photos were taken of informative structures from herbarium specimens (e.g., leaves, fruits, bark samples, etc.) using the high-resolution imaging equipment at the MNHN in Paris (a Nikon D7100 camera with a Nikkor AF-S 60 mm macro lens mounted on a Kaiser light stand equipped with a Cognisys StackShot Macro rail). Two methods were employed. For two-dimensional objects such as leaves, photos were taken using the macro mode in the Nikon Camera Control software (ver. 2.0), whereas for large objects such as fruits or those with significant relief (e.g., bark), z-stacking was done using the Helicon Remote program (ver. 3.9.12) and Helicon Focus (ver. 8.1.1), which creates a final image with unlimited depth of field by electronically superimposing the in-focus portions of a series of shots taken at distinct levels and combining them into a single image that clearly shows the entire structure being photographed. A total of ca. 4,500 images were taken from 200 bark samples representing 40 of the 88 large tree species of *Diospyros* (bark material was not available for the other 48 species).

An integrated glossary provides quick access to clear definitions of all technical terms, often accompanied by photos and/or illustrations. The definitions presented to the user were compiled from several widely used sources, including Beentje (2016) and Harris and Harris (2001) for general terminology, and Ellis et al. (2009) for features relating to leaf architecture. The utility of the *Diospyros* key was further strengthened by providing links to internet sources for each species, including the corresponding pages in the Madagascar Catalogue (2024) and the IUCN Red List (IUCN 2024), although these can only be accessed when connected to the internet. However, because precise information on the distribution of precious wood species is potentially sensitive, access to maps and full data on known occurrences is limited in these on-line sources and is not available to the general public.

#### ﻿Testing the key and integrating feedback

Early iterations of the *Diospyros* key underwent thorough evaluation and testing by botanists familiar with Madagascar’s precious woods, particularly *Diospyros*, as well as by non-specialists. This was done during a series of working sessions in which participants provided feedback through evaluation forms. The results were utilized to enhance and fine-tune both the structure and content of the key. Subsequently, three initial trial identification workshops using herbarium specimens were held (in June and September 2021, and October 2022) for groups of participants with varying levels of knowledge and experience. The main objectives were to:

1. Review and discuss the multi-access structure of the key and ease of interpretation of the characters.
2. Determine which of the morphological and eco-geographic characters were the most informative for making accurate species-level identifications.
3. Test the overall performance of the key and identify problems or gaps.

In April 2022, an additional test workshop was conducted for students participating in the Madagascar precious woods project at the University of Antananarivo, and six regional training workshops were organized between November 2022 and August 2023 for various important stakeholder groups such as customs agents, members of the judiciary police, police officers, forestry agents, and conservation site managers. The purpose of these workshops was to introduce the key to the participants and gather their comments and suggestions.

## ﻿Results

### ﻿Key efficacy, character performance, and limits

During the initial test sessions, participants were able to achieve correct identifications from 65–75% of the time. The feedback received after each workshop was then used to make improvements to the *Diospyros* key. Specifically, adjustments were made regarding the organization and structure of characters and character states. The presentation to the user was also modified based on the logical progression from basal to apical organs and from macro- to microscopic features. Additionally, the characters were re-categorized into two main groups within the key, “Morphology” and “EcoGeo”.

**Figure 5.**
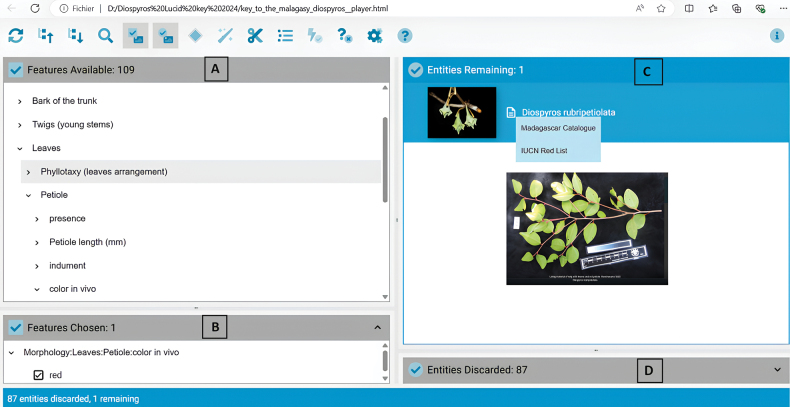
Screenshot of the deployed key in the Lucid player interface showing use of the characters that are most obvious when examining a sample with the naked eye. The example shown here assumes that the key had already been used to identify a sample taken from a tree in the field whose leaves have a red petiole **A** by selecting the character state “red” for petiole color *in vivo*, a single candidate species is retained **B** window showing the number of selected features and the chosen path **C** window showing the number of remaining entities after one or more characters have been selected, in this case, the single entity corresponding to *Diospyrosrubripetiolata*; the image viewer can be accessed by clicking on the first image displayed **D** window indicating the number of discarded entities; in this case, the 87 species that did not meet the selected identification criterion ‘red petioles *in vivo*’.

The results obtained from the trials indicated that the initial versions of the key were highly successful at leading users to the correct identification. The fact that the key is designed to allow multi-access utilization was clearly a strength because it enabled users to follow their intuition when selecting the order of characters to be entered. The botanists who tested the key made several suggestions for its improvement and for how it can be used most efficiently. They recommended that a user first note any obvious and striking features of the material being identified. For example, one of the most evident characters in fresh material of *Diospyrosrubripetiolata* is the red tinge of the petiole, and if the user goes directly to the ‘features’ window and selects Morphology/Leaves/Petiole/Color_in_vivo/Red, the number of ‘discarded entities’ that do not express this particular character state (which is indicated in the lower left of the window) is indicated as 87, while the number of ‘remaining entities’ is indicated as 1 in the upper right, corresponding to *D.rubripetiolata* (Fig. 5). By clicking on the first photo in the species profile, the ‘image viewer’ displays all the images corresponding to the species, several of which clearly show the red petioles, thereby rapidly facilitating verification of the identification.

The second suggestion made during the test phase was that, in the absence of obvious and informative morphological characters, the user should start by focusing on eco-geographic parameters. In particular, they should begin by entering the geographic coordinates (longitude and latitude) of the location where the sample was obtained, which provides an efficient way to reduce the number of candidate species. For example, when one enters the coordinates corresponding to the 1°×1° cell for localities with a longitude starting with 46°E and a latitude starting with 15°S (using the format called for in the key, ‘4615’), 74 entities are excluded and only 14 candidate species are retained (i.e., just 16% of the 88 large tree species of *Diospyros* occurring in Madagascar).

A third suggestion was that, when using vegetative characters, leaf features should be considered first because they are often the most effective for distinguishing species from one another. For example, the cordate leaf shape of *Diospyrosvescoi* combined with the presence of indument on both surfaces of the leaf blade are uniquely diagnostic for this species. When reproductive organs are available, the fruit is often useful and informative, especially features of the fruiting calyx, such as the degree to which it covers the fruit surface and the presence/absence of lobes. As an example, if the initial entry of characters indicates *D.mapingo* and *D.tropophylla* as the two remaining candidate species, they can easily be distinguished based respectively on the presence or absence of lobes on the fruiting calyx.

Regarding characters seen as difficult to interpret or not particularly useful or pertinent for identification in the field or the herbarium (and thus rarely chosen during testing), the participants mentioned that subjective or ambiguous features requiring the user to make a personal interpretation, such as color (for both fresh or dried material) and leaf texture, were frequently scored differently by different persons. Likewise, it can be challenging to select the correct character state for features such as trichome type and length, which often requires using a hand lens or microscope, and is thus not always possible or practical in the field, especially for someone who is not familiar with the corresponding technical terminology. These characters are therefore more appropriate for identifying herbarium specimens and for use by experienced botanists.

After incorporating user feedback and remarks to improve the overall accuracy, appearance, and user-friendliness of the *Diospyros* key, it is now fully operational, enabling error-free identification of species 90–100% of the time.

Several practical aspects should be considered when developing and refining Lucid keys such as the one we have prepared for Malagasy *Diospyros*. The use of English could be an issue for some users not intimately familiar with terminology in this language. Several participants suggested during the testing phase that it would be helpful to have a version in French or even in Malagasy (which would be more challenging to develop as many technical terms do not exist in this language). It would also be helpful to have a version developed for use on smartphones, which are more portable and would facilitate use at remote field sites lacking internet access. Conversion of the Lucid tool into a mobile application for Android or iOS can be done, but publication of a fully functional key would involve an additional 5-step development phase requiring paid services that can only be provided by the Lucid team.

## ﻿Conclusions and perspectives

As part of a coordinated effort to promote the sustainable and equitable use of precious woods resources in Madagascar in response to the CITES Action Plan regarding the genera *Diospyros* and *Dalbergia*, and in particular with respect to the development of practical and reliable identification tools for species that are of potential commercial interest, we have developed a key using Lucid that can be employed to identify standing and recently felled trees as well as herbarium specimens. This powerful, interactive, multi-access key can be used without an internet connection and is accessible to a broad group of users, ranging from non-specialists to experienced botanists. Moreover, since most trees encountered during forest inventories lack flowers and fruits, the key was designed specifically to enable accurate identification of sterile material using characters that can be observed in the field throughout the year. Special attention was given to incorporating vegetative characters, including bark features, which have been shown to be useful and informative for distinguishing *Diospyros* species in the field (further work is being conducted to explore the taxonomic value of bark characters). Eco-geographic features were also used to develop the key and were found to be particularly valuable for increasing the speed and accuracy of identifications by returning only candidate species that are known or inferred to occur within a given 1°×1° cell.

The morphological and eco-geographic characters employed in our key enable accurate identification of all 88 species of *Diospyros* in Madagascar that form large enough trees to be potential sources of commercially valuable ebony wood. Each species is accompanied by its own set of images to help verify initial identifications, along with links to additional information available online, and most technical terms are likewise illustrated to facilitate accurate comprehension and interpretation. Lucid is a tried and tested platform that is flexible and offers many useful options, enabling regular improvements and updates, including the addition of new species and informative characters. For example, as work progresses on developing a comprehensive understanding of characters based on anatomy, spectroscopy, and even DNA barcoding of Malagasy *Diospyros*, taxonomically informative features can be incorporated into our key. As part of the G3D project focusing on precious woods in Madagascar, a key has also been developed for the 56 large tree species of *Dalbergia* from which rosewood and palisander are obtained.

There remain some aspects that could be refined for further improvement of the current version of the key. Additional field testing encompassing multiple populations of each species (especially those that exhibit significant morphological variation) would be valuable. This could improve our knowledge of character variation, add new features while removing those that are less informative or more difficult to interpret, and fill gaps in character states for some species (notably for bark features), identifying and correcting errors that may have inadvertently been introduced into the character data matrix. After an ongoing round of improvements now being completed, as part of the second phase of the G3D project, the key will be translated into French to render it more accessible for francophone users, which should be finalized during 2025. In parallel, we will seek support to develop and test a stand-alone, portable version for smartphones. Lucid’s flexibility and wide range of functionality make it possible to design and develop practical, multi-access digital keys that can easily be updated as new information becomes available. Finally, the methods and approach used to develop this practical identification tool for potentially exploitable Malagasy *Diospyros* species could easily be expanded to include all ca. 285 members of the genus occurring on Madagascar and all 83 species of *Dalbergia* (Madagascar Catalogue 2024) as well as to other groups (genera and even families) of plants and animals of commercial and/or scientific interest.

### ﻿Key security and accessibility

Because many of the *Diospyros* species harvested in Madagascar as sources of ebony wood are threatened by over-exploitation and illegal logging, information relating to their characterization and distribution is potentially sensitive. To ensure that access to and use of the prototype key presented here for identifying large tree species can be adequately controlled, we have opted to host it on a password protected server (https://www.mobot.org/sci/), making it available to appropriate users when a request is sent to the first author. Upon obtaining a password, the user will be able to download a stand-alone version of the key or access an html link that can be opened on any web browser, both online or offline.

## ﻿Additional information

### Author ORCIDs

Hasina N. Rakouth https://orcid.org/0009-0004-8279-5245

Bakolimalala Rakouth https://orcid.org/0000-0001-5710-2006

Peter B. Phillipson https://orcid.org/0000-0001-8390-0079

Porter P. Lowry II https://orcid.org/0000-0002-8047-5019

Nicholas Wilding https://orcid.org/0000-0003-4029-5387

### Data availability

All of the data that support the findings of this study are available in the main text or Supplementary Information.

## ﻿Appendix 1

**Table A1.** The 88 large tree species included in the *Diospyros* key (in alphabetical order) and their IUCN Red List status. CR: Critically endangered; DD: Data Deficient; EN: Endangered; LC: Least Concern; NT: Near Threatened; VU: Vulnerable (from Lowry et al. 2024).

Species	IUCN Red List status
*Diospyrosaculeata* H. Perrier	LC
*Diospyrosambanjensis* G.E. Schatz & Lowry	EN
*Diospyrosamborelloides* G.E. Schatz & Lowry	VU
*Diospyrosanalamerensis* H. Perrier	EN
*Diospyrosandohahelensis* G.E. Schatz & Lowry	CR
*Diospyrosankaranensis* Mas, G.E. Schatz & Lowry, ined.	EN
*Diospyrosantakaranae* Capuron ex G.E. Schatz & Lowry	VU
*Diospyrosantongilensis* G.E. Schatz & Lowry	NT
*Diospyrosantsiranana* G.E. Schatz & Lowry	VU
*Diospyrosarenicola* A.G. Linan, G.E. Schatz & Lowry	VU
*Diospyrosbardotiae* H.N. Rakouth, G.E. Schatz & Lowry	VU
*Diospyrosbaroniana* H. Perrier	LC
*Diospyrosbaronii* (H. Perrier) H.N. Rakouth & Lowry	NT
*Diospyrosbeberonnii* G.E. Schatz & Lowry	EN
*Diospyrosbemarivensis* H. Perrier	VU
*Diospyrosbernieriana* (Baill.) H. Perrier	LC
*Diospyrosbezofensis* H. Perrier	EN
*Diospyrosbrevipedicellata* G.E. Schatz, Lowry & Mas, ined.	LC
*Diospyroscalophylla* Hiern	LC
*Diospyroschitoniophora* Capuron ex A.G. Linan, G.E. Schatz & Lowry	VU
*Diospyrosclusiifolia* (Hiern) G.E. Schatz & Lowry	NT
*Diospyroscrassifolia* A.G. Linan, G.E. Schatz & Lowry	VU
*Diospyroscrassipedicellata* G.E. Schatz & Lowry	VU
*Diospyroscupulifera* H. Perrier	LC
*Diospyrosdecaryoides* G.E. Schatz & Lowry	VU
*Diospyrosdolichopoda* G.E. Schatz, Lowry & Mas, ined.	VU
*Diospyrosebenifera* (H. Perrier) G.E Schatz & Lowry	EN
*Diospyroserythrosperma* H. Perrier	LC
*Diospyrosfalyi* G.E. Schatz & Lowry	EN
*Diospyrosfuscovelutina* Baker	NT
*Diospyrosgracilipes* Hiern	LC
*Diospyrosgrandiflora* G.E. Schatz & Lowry	EN
*Diospyroshaplostylis* Boivin ex Hiern	LC
*Diospyroshazomainty* H. Perrier	DD
*Diospyroshumbertiana* H. Perrier	LC
*Diospyrosimplexicalyx* H. Perrier	EN
*Diospyroslabatiana* Mas, G.E. Schatz & Lowry, ined.	LC
*Diospyroslanceolata* Poir.	NT
*Diospyroslewisiae* Mas, G.E. Schatz & Lowry, ined.	VU
*Diospyroslittoralis* Capuron ex G.E. Schatz & Lowry	VU
*Diospyroslowryi* G.E. Schatz	EN
*Diospyrosmaculata* G.E. Schatz & Lowry	LC
*Diospyrosmadagascariensis* (A.DC.) E. Mestre & H.N. Rakouth	LC
*Diospyrosmahaboensis* G.E. Schatz, Lowry & Mas, ined.	EN
*Diospyrosmalandy* H.N. Rakouth, G.E. Schatz & Lowry	EN
*Diospyrosmanampetsae* H. Perrier	LC
*Diospyrosmandenensis* H.N. Rakouth, G.E. Schatz & Lowry	VU
*Diospyrosmapingo* H. Perrier	LC
*Diospyrosmasoalensis* H. Perrier	NT
*Diospyrosmeeusiana* (H. Perrier) G.E. Schatz & Lowry	EN
*Diospyrosmelanocarpa* G.E. Schatz & Lowry	VU
*Diospyrosmicrogracilipes* G.E. Schatz, Lowry & Mas, ined.	EN
*Diospyrosmimusops* G.E. Schatz & Lowry	EN
*Diospyrosmyriophylla* (H. Perrier) G.E. Schatz & Lowry	LC
*Diospyrosmyrtifolia* H. Perrier	LC
*Diospyrosocclusa* H. Perrier	LC
*Diospyrosolacinoides* (H. Perrier) G.E. Schatz & Lowry	LC
*Diospyrosolivieri* A.G. Linan, G.E. Schatz & Lowry	CR
*Diospyrosorbicularis* G.E. Schatz & Lowry	VU
*Diospyrosparifolia* H. Perrier	NT
*Diospyrosparvifolia* Hiern	LC
*Diospyrosperrieri* Jum.	NT
*Diospyrosplatycalyx* Hiern	LC
*Diospyrosplicaticalyx* A.G. Linan, G.E. Schatz & Lowry	EN
*Diospyrospubiramulis* A.G. Linan, G.E. Schatz & Lowry	VU
*Diospyrosquadrangularis* G.E. Schatz & Lowry	LC
*Diospyrosrakotovaoi* G.E. Schatz & Lowry	VU
*Diospyrosramisonii* G.E. Schatz & Lowry	VU
*Diospyrosrandrianasoloi* G.E. Schatz, Lowry & Mas, ined.	LC
*Diospyrosranirisonii* G.E. Schatz, Lowry & Phillipson	VU
*Diospyrosretusa* H.N. Rakouth, G.E. Schatz & Lowry	EN
*Diospyrosrubripetiolata* G.E. Schatz & Lowry	LC
*Diospyrossakalavarum* H. Perrier	LC
*Diospyrossclerophylla* H. Perrier	VU
*Diospyrossennenii* G.E. Schatz & Lowry	LC
*Diospyrossphaerosepala* Baker	NT
*Diospyrossquamosa* Bojer ex A. DC.	LC
*Diospyrossubenervis* (H. Perrier) G.E. Schatz & Lowry	VU
*Diospyrossubtrinervis* H. Perrier	CR
*Diospyrostaikintana* G.E. Schatz & Lowry	CR
*Diospyrostampolensis* H.N. Rakouth, G.E. Schatz & Lowry	CR
*Diospyrostorquata* H. Perrier	NT
*Diospyrostoxicaria* Hiern	LC
*Diospyrostropophylla* (H. Perrier) G.E. Schatz & Lowry	LC
*Diospyrosultima* G.E Schatz & Lowry	EN
*Diospyrosurschii* H. Perrier	NT
*Diospyrosvelutipes* (H. Perrier) G.E. Schatz & Lowry	LC
*Diospyrosvescoi* Hiern	LC

## ﻿Appendix 2

**Table A2.** Eco-geographic and morphological available features, with the 109 characters and 356 character states used in the Lucid key for Malagasy *Diospyros*.

“**Eco-geography” or Eco-geographic available features**		
**Features**	**Characters**	**Character states**
**Eco-geography**	Bioclimate	Humid, Subhumid, Dry, Subarid, Montane
	Elevation (m)	-499, -999, -1499, -1999, -2499
	Longitude E/ Latitude S coordinates coded with 4 digit number	Numerical values (#). The first pair of digit corresponds to the degree longitude, and the second pair to the degree latitude. For example: if the relevant coordinates are **16**° 77719 S and **46**° 85432 E or **16**°46'37"S and **46**°51'15"E, the observed value should be entered as (longitude first and latitude second) : **4616**
“**Morphology” or Morphological available features**		
**Organs features**	**Characters**	**Character states**
**WHOLE PLANT**	Growth form	Shrub (woody, 1–7 m tall), tree over 7 m tall, usually single stem
**BARK OF THE TRUNK**	Surface texture	Smooth, fissured (=cracked), flaky (rhytidom), rugose
	Structure present	Lenticels, blisters, elongate striations, transverse striations, flakes, shallow cervices
	**External bark**	
	Color. *In vivo*	white/whitish, gray/grayish, light brown, dark brown, black/blackish
	Color. *In sicco*	white/whitish, gray/grayish, light brown, dark brown, black/blackish
**TWIGS (YOUNG STEMS)**	Cross section	Terete, flattened, square
	Surface structures on twigs bark	Lenticels, blisters, elongate striations, transverse striations, fissures or cracks (including microfissure), flakes/thin flakes, micro-spines
	**Indument on twigs (young stems)**	
	White waxy substance. *Presence*	Absent, present
	Trichomes (hairs). *Presence*	Absent, present
	Trichomes (hairs). *Persistence*	Persistent, not persistent
	Trichomes (hairs). *Density*	Dense, moderately dense, sparse
	Trichomes (hairs). *Orientation*	Erect, semi-erect, semi appressed, appressed, curly
	Trichomes (hairs). *Length* (in mm)	Numerical values (#)
	Trichomes (hairs). *Color*	White/whitish, gray/grayish, brown/ light brown, golden/yellowish, tawny (yellowish brown)/ferruginous, orange/reddish brown
	Twigs. Color (*in sicco*)	White/whitish/light gray, brownish-green, brown/light brown, dark brown/ reddish-brown, dark gray, black/blackish
**LEAVES**	Phyllotaxy	Alternate, spiral, opposite (subopposite), whorled (verticillate)
**PETIOLE**	Presence	Absent (leaf sessile), present (leaf petiolate)
	If present. *Length* (in mm)	Numerical values (#)
	**Indument on petiole**	
	White waxy substance. *Presence*	Absent, present
	Trichomes (hairs). *Presence*	Absent, present
	Trichomes (hairs). *Persistence*	Persistent, not persistent
	Trichomes (hairs). *Density*	Dense, moderately dense, sparse
	Trichomes (hairs). *Orientation*	Erect, semi-erect, semi appressed, appressed, curly
	Trichomes (hairs). *Length* (in mm)	Numerical values (#)
	Indument. Trichomes (hairs). *Color*	White/whitish/light gray, brown/ light brown, golden/yellowish, tawny (yellowish brown)/ferruginous, orangish/reddish brown
	Color of the petiole. *In vivo*	Green, yellowish-green, brownish-green, light (pale) brown, red, glaucous
	Color of the petiole. *In sicco*	Gray/grayish/glaucquescent, brownish-green, light brown/yellowish-brown, dark brown/reddish, black/blackish
**Morphology**		
**Features**	**Characters**	**Character states**
**LAMINA (LEAF BLADE)**	Texture	Coriaceous, subcoriaceous, chartaceous, membranaceous
	Shape	Elliptic to largely elliptic, oblong, ovate, obovate, oblanceolate, cordate, orbicular, lanceolate
	Dimensions of the largest leaf. *Length* (in cm)	Numerical values (#)
	Dimensions of the largest leaf. *Width* (in cm)	Numerical values (#)
	**Leaf base**	
	*Shape*	Attenuate/decurrent, cuneate, acute, obtuse, rounded, truncate, subcordate/cordate
	*Symmetry of the base in relation to the midvein*	Symmetric, asymmetric
	Margin	Flat (thin), revolute, minutely revolute, undulate, slightly undulate, slightly thickened on the leaf blade below surface
	**Apex**	
	Mucron presence	Absent, present
	Shape	Acute, acuminate, obtuse, rounded, emarginate/retuse;
	**Acumen of the apex**	
	Presence	Absent, present
	if present, *length* (in mm)	Numerical values (#)
	Shape of the acumen tip	Retuse, apiculate, rounded, acute
	**Primary vein/ midvein**	
	Upper surface	Distinctly raised, flat, slightly impressed, sunken/impressed or distinctly depressed
	Lower surface	Distinctly raised, slightly raised, flat
	**Secondary veins. Upper surface**	
	Visibility/appearance	Obscure/indistinct, weakly visible, raised, slightly raised, flat, sunken/impressed,
	Number per side	Numerical values (#)
	Tertiary veins /intersecondaries presence	Absent, present
	**Secondary veins. Lower surface**	
	Visibility/appearance	Obscure/indistinct, weakly visible, raised, slightly raised, flat, sunken/impressed
	Venation type	Brochidodromous, craspedodromous, semicraspedodromous, reticulodromous, eucamptodromous, cladodromous
	**Indument on leaf blade. Upper surface**	
	White waxy substance. *Presence*	Absent, present
	Trichomes (hairs). *Presence*	Absent, present
	Trichomes (hairs). *Persistence*	Persistent, not persistent
	Trichomes (hairs). *Density*	Dense, moderately dense, sparse
	Trichomes (hairs). *Disposition*	Throughout the lamina except on venation, all over venation, on midvein only, on the margin or on the edge of leaf blade
	Trichomes (hairs). *Orientation*	Erect, semi-erect, semi-appressed, appressed, irregular/messy or confused/curly
	Trichomes (hairs). *Length* (in mm)	Numerical values (#)
	Indument. Trichomes (hairs). *Color*	White/whitish, gray/grayish (silvery), brown/ light (pale) brown, golden/yellowish, tawny/fauve/orange (yellowish-brown)/ferruginous, brown/reddish brown
	**Indument on leaf blade venation. Lower surface**	
	White waxy substance. *Presence*	Absent, present
	Trichomes (hairs). *Presence*	Absent, present
	Trichomes (hairs). *Persistence*	Persistent, not persistent
	Trichomes (hairs). *Density*	Dense, moderately dense, sparse
	Trichomes (hairs). *Disposition*	Throughout the lamina except on venation, all over venation, on midvein only, on the margin or on the edge of leaf blade
**Morphology**		
**Features**	**Characters**	**Character states**
**LAMINA (LEAF BLADE)**	Trichomes (hairs). *Orientation*	Erect, semi-erect, semi-appressed, appressed, irregular/messy or confused/curly
	Trichomes (hairs). *Length* (in mm)	Numerical values (#)
	Indument. Trichomes (hairs). *Color*	White/whitish/gray, brown/ light (pale) brown, dark-brown/brown, golden/yellowish, tawny/fauve/orange (yellowish-brown)/ferruginous, reddish brown
**FEMALE FLOWERS**	Position and place of birth	Axillary, borne on twigs (ramiflorous), on stem (cauliflorous), on trunk (trunkiflorous), or on short shoots (on brachyblasts)
	Inflorescence types	Solitary, born in 2 s or 3 s, glomerules, fascicules, cymes, umbels, or branched/multiple
**PERSISTENT FRUITING CALYX**	Degree of enclosure of the fruit	Fully enclosed (100% fruit surface obscured), largely enclosed (at least 50%), partially enclosed (< 50%)
	Total height (incl. lobes if present)	Height (in mm) Numerical values (#)
	**Calyx lobes**	
	Presence	Absent (calyx entire), present (calyx lobed)
	Number	3, 4, 5, 6
	Degree of fusion	Partially united, entirely united (calyx accrescent at the base), entirely free (rare)
	Dimensions. *Length* (in mm)	Numerical values (#)
	Dimensions. *Width* (in mm)	Numerical values (#)
	Shape	Oblong, elliptic, obovate, semi-ovate warhead shaped, orbicular, triangular, linear
	Orientation	Erect, spreading, reflexed
	Margins (shape)	Flat, revolute, minutely revolute, involute, plicate-undulate, lacerate
	Apex (shape)	Acute, obtuse, rounded, retuse
	Venation, striations presence	Absent (indistinct), present (distinct)
	**Calyx indument. Outer surface**	
	White waxy substance. *Presence*	Absent, present
	Trichomes (hairs). *Presence*	Absent, present
	Trichomes (hairs). *Persistence*	Persistent, not persistent
	Trichomes (hairs). *Density*	Dense, moderately dense, sparse
	Trichomes (hairs). *Disposition*	Throughout, apical portion, basal portion
	Trichomes (hairs). *Orientation*	Erect, semi-erect, semi appressed, appressed, curly); color
	Trichomes (hairs). *Length* (in mm)	Numerical values (#)
	Trichomes (hairs). *Color*	White/whitish, gray/grayish, brown/ light brown, brownish-orange/ reddish brown, golden/yellowish, fauve/orange/tawny (yellowish brown)/ferruginous/rusty, dark-brown/blackish
	**Calyx indument. Inner surface**	
	White waxy substance. *Presence*	Absent, present
	Trichomes (hairs). *Presence*	Absent, present
	Trichomes (hairs). *Persistence*	Persistent, not persistent
	Trichomes (hairs). *Density*	Dense, moderately dense, sparse
	Trichomes (hairs). *Disposition*	Throughout, apical portion, basal portion
	Trichomes (hairs). *Orientation*	Erect, semi-erect, semi appressed, appressed, curly
	Trichomes (hairs). *Length* (in mm)	Numerical values (#)
	Trichomes (hairs). *Color*	White/whitish, gray/grayish, brown/ light brown, brownish-orange/ reddish brown, golden/yellowish, fauve/orange/tawny (yellowish brown)/ferruginous/rusty, dark-brown/blackish
**FRUIT BODY**	Shape	Ellipsoid, obloid, ovoid, spherical (globose), subspherical (subglobbose)
	Height (in mm)	Numerical values (#)
	Apex shape	Acute, apiculate/rostrate, rounded, flat/depressed
	External texture	Smooth and matte, smooth and shiny, rugose/rough, verrucose (warty), pubescent
**Morphology**		
**Features**	**Characters**	**Character states**
**FRUIT BODY**	**Indument on the fruit body**	
	White waxy substance. *Presence*	Absent, present
	Trichomes (hairs). *Presence*	Absent, present
	Trichomes (hairs). *Persistence*	Persistent, not persistent
	Trichomes (hairs). *Density*	Dense, moderately dense, sparse
	Trichomes (hairs). *Disposition*	Throughout, apical portion, basal portion
	Trichomes (hairs). *Orientation*	Erect, semi-erect, semi appressed, appressed, curly
	Trichomes (hairs). *Length* (in mm)	Numerical values (#)
	Trichomes (hairs). *Color*	White/whitish, gray/grayish, brown/ light brown, brownish-orange/ reddish brown, golden/yellowish, fauve/orange/tawny (yellowish brown)/ferruginous/rusty, dark-brown/blackish
	Color of the fruit body. *In vivo*	Green, yellow/yellowish-green, orange, red, light brown/beige, white (due to white waxy substance), dark brown;
	Color of the fruit body. *In sicco*	Light brown/yellowish-brown, white (due to persistent white waxy substance), dark brown/ferruginous, black blackish

## References

[B1] Andriamanana DJ (2019) Gouvernance du secteur forestier à Madagascar: analyse du mécanisme de prise de décision dans le cadre de la lutte contre le trafic de bois précieux. Thèse de doctorat en Sciences Agronomiques et Environnementales, École Supérieure des Sciences Agronomiques, Université d’Antananarivo. Antananarivo.

[B2] Andriambololonera S Andrianarivelo S Ravololomanana N Randriambololomamonjy O (2013) Rapport de l’étude sur les taxons de bois précieux *Diospyros* spp. et *Dalbergia* spp., en vue de leur inscription dans l’annexe II de la CITES. Missouri Botanical Garden, 16.

[B3] Ballet J Lopez P Rahaga N (2011) L’ exportation de bois précieux (*Dalbergia* et *Diospyros*) « illégaux » de Madagascar: 2009 et après? Madagascar Conservation and Development 5(2): 110–116. 10.4314/mcd.v5i2.63141

[B4] Beentje H (2016) The Kew plant glossary: an illustrated dictionary of plants. Second edition. Kew Publishing, Royal Botanic Gardens, Kew, 184.

[B5] Begum MM Dalisay TU Cumagu CJ (2012) Taxonomic review of and development of a Lucid key for Philippine Cercosporoids and related fungi. Chapter I. Plant Pathology, 1–40. 10.5772/30214

[B6] Biswas S Gupta K Talapatra SN (2016) A digitized database of bark morphology for identification of common tree species and literature study of bark phytochemicals and therapeutic usage. World Scientific News 42: 143–155. https://web.archive.org/web/20180423011045id_/http://www.worldscientificnews.com/wp-content/uploads/2015/10/WSN-42-2016-143-1551.pdf

[B7] Carpentier M Giguere P Gaudreault J (2018) Tree species identification from bark images using convolutional neural networks. IEEE International Conference on Intelligent Robots and Systems (IROS), 1075–1081. 10.1109/IROS.2018.8593514

[B8] CITES (1978) Convention on International Trade in Endangered Species of wild fauna and flora. Environmental Policy and Law 4: 51–52. 10.1016/S0378-777X(78)80178-4 712806

[B9] CITES (2013a) Convention on International Trade in Endangered Species of wild fauna and flora. CoP16 Prop. 58. Consideration of proposals for amendment of Appendices I and II. https://www.cites.org:sites/default/files/eng/cop/16/prop/E-CoP16-Prop-58.pdf

[B10] CITES (2013b) Convention on International Trade in Endangered Species of wild fauna and flora. CoP16 Prop. 63. Consideration of proposals for amendment of Appendices I and II. https://www.cites.org:sites/default/files/eng/cop/16/prop/E-CoP16-Prop-63.pdf

[B11] CITES (2018) Convention on International Trade in Endangered Species of wild fauna and flora. PC24 Doc. 18.2. Species specific matters: Malagasy ebonies (Diospyros spp.) and palisanders and rosewoods (*Dalbergia* spp.). Report of the Secretariat. Decision 17.208. https://cites.org/sites/default/files/eng/com/pc/24/E-PC24-18-02.pdf

[B12] CITES (2024) Convention on International Trade in Endangered Species of wild fauna and flora. PC27. Doc.41.4. Annex 1. CITES *Diospyros* checklist: population of Madagascar, large tree species. First edition. www.cites.org

[B13] Cornet A (1974) Essai de cartographie bioclimatique à Madagascar (No. 55). Orstom, Paris, 28. https//www.documentation.ird.fr/hor/fdi:06946

[B14] Crameri S Phillipson PB Rakotonirina N Wilding N Andriamiarisoa RL Lowry PP II Widmer A (2022) Taxonomic studies on Malagasy *Dalbergia* L. (Fabaceae). III. Two new species from southeastern Madagascar and an emended description of the rosewood species *Dalbergiamaritima*. Systematic Botany 47(2): 397–416. 10.1600/036364422X16512564801614

[B15] Dallwitz MJ (1993) DELTA and Intkey. Advances in computer methods for systematic biology: artificial intelligence, databases, computer vision. The Johns Hopkins University Press, Baltimore, Maryland, 287–296. http://delta-intkey.com/www/dallwitz-1993-delta_intkey.htm

[B16] Dallwitz M (2007) A comparison of interactive identification programs. http://www.delta-test1.com/www/comparison.pdf

[B17] Delaunay M (2020) Comment faciliter l’identification de l’entomofaune? Construction, évaluation et amélioration de clés d’identification numériques. Doctoral dissertation, Museum national d’histoire naturelle-MNHN Paris. https://theses.hal.science/tel-02868459

[B18] Ellis B Daly DC Hickey LJ Johnson KR Mitchell JD Wilf P Wing SL (2009) Systematic botany. Manual of leaf architecture. Cornell University Press, Ithaca, New York, 825–825. 10.1600/036364409790139682

[B19] Gaubert P Chalubert A Dubus G (2008) An interactive identification key for genets and oyans (Carnivora, Viverridae, Genettinae, *Genetta* spp. and *Poiana* spp.) using Xper2. Zootaxa 1717(1): 39–50. 10.11646/zootaxa.1717.1.4

[B20] Global Witness & Environmental Investigation Agency EIA (2009) Mission d’investigation, trafic de bois précieux à Madagascar. Rapport, 19.

[B21] Global Witness & Environmental Investigation Agency EIA (2010) Rapport d’enquête sur le commerce mondial des bois précieux malgaches: bois de rose, ébène et palissandre, 32.

[B22] Griffing LR (2011) Who invented the dichotomous key? Richard Waller’s watercolors of the herbs of Britain. American Journal of Botany 98(12): 1911–1923. 10.3732/ajb.1100188 22074776

[B23] Hagedorn G Ram G Martellos S (2010) Types of identification keys. In: Nimis PL Lebbe RV (Eds) Tools for identifying biodiversity: progress and problems.Proceedings of the International Congress, Paris. Edizioni Università di Trieste, 59–64.

[B24] Hansen B Rahn K (1969) Determination of angiosperm families by means of a punched-card system. Dansk Botanisk Arkiv 26(1): 7.

[B25] Hardisty A Roberts D (2013) A decadal view of biodiversity informatics: challenges and priorities. BMC Ecology 13: 1–23. 10.1186/1472-6785-13-16 23587026 PMC3843378

[B26] Harris JG Harris WM (2001) Plant identification terminology. An illustrated glossary, 2^nd^ edn. Spring Lake Publishing, Spring Lake, Utah.

[B27] Hassler M (2023) World Plants. Synonymic checklist and distribution of the world flora. Version 18.3. https://www.worldplants.de

[B28] Hickey L Ash A Ellis B Johnson K Wilf P Wing S (1999) Manual of leaf architecture, 65 pp.

[B29] Innes J (2010) Madagascar rosewood, illegal logging, and the tropical timber trade. Madagascar Conservation and Development 5(1): 5–10. 10.4314/mcd.v5i1.57335

[B30] IUCN International Union for Conservation (2024) The IUCN Red list of threatened Species website version 2024-2. https://www.iucnredlist.org/species [accessed 18.05.2024]

[B31] IUCN SSC International Union for Conservation of Nature Species Survival Commission (2012) IUCN Red List categories and criteria, version 3. 1, 2nd edn. Gland and Cambridge, 32 pp.

[B32] IUCN SSC International Union for Conservation of Nature Species Survival Commission (2024) Guidelines for Using the IUCN Red List Categories and Criteria, version 16. Prepared by the Standards and Petitions Committee. https://www.iucnredlist.org/documents/RedListGuidelines.pdf

[B33] Judd W Campbell S Kellogg A Stevens E (1999) Plant Systematics- A phylogenetic approach. Inc. Publishers, Sunderland, 465.

[B34] Judd WS Campbell CS Kellogg EA Stevens PF Donoghue MJ (2002) Plant Systematics. A phylogenetic approach, 2^nd^ edn. Sinauer S. Sunderland, Massachusetts, 576 pp.

[B35] Junikka L (1994) Survey of English macroscopic bark terminology. IAWA Journal 15(1): 3–45. 10.1163/22941932-90001338

[B36] Juola J Hovi A Rautiainen M (2022) Classification of tree species based on hyperspectral reflectance images of stem bark. European Journal of Remote Sensing 56(1): 1–15. 10.1080/22797254.2022.2161420

[B37] Letouzey R-G (1969) Manuel de botanique forestière. Afrique Tropicale. Tome 1. Nogent sur Marnes. Centre Technique Forestier Tropical, 189 pp.

[B38] Linan AG Lowry PP II Schatz GE (2021) Taxonomic studies of *Diospyros* (Ebenaceae) from the Malagasy region. VII. Revision of DiospyrosSect.Forbesia in Madagascar and the Comoro Islands. Annals of the Missouri Botanical Garden 106: 72–110. 10.3417/2021644

[B39] Linan AG Rakouth HN Rabarimanarivo M Schatz GE Lowry PP II (2024) Taxonomic studies of *Diospyros* (Ebenaceae) from the Malagasy region. XI. Revision of the Tetraclis group. Candollea 79(1): 129–169. 10.15553/c2024v791a8

[B40] Lombard N le Roux MM van Wyk BE (2021) Electronic identification keys for species with cryptic morphological characters: A feasibility study using some *Thesium* species. PhytoKeys 172: 72–119. 10.3897/phytokeys.172.53484 PMC7902595 33664610

[B41] Lowry PP II Schatz GE Leroy J-F Wolf A-E (1999) Endemic families of Madagascar. III. A synoptic revision of *Schizolaena* (Sarcolaenaceae). Adansonia 3: 183–212.

[B42] Lowry PP II Phillipson BP Rakouth HN Andriambololonera S Rabarimanarivo M Manjato N (2024) Large tree species of *Diospyros* from Madagascar. Species that are known or suspected to produce wood of commercial value. http://www.tropicos.org/NamePage.aspx?nameid=40031908&projectid=17

[B43] Madagascar Catalogue (2024) Catalogue of the Plants of Madagascar. Missouri Botanical Garden, St Louis, MO and Tropicos, Antananarivo. http://legacy.tropicos.org/projectwebportal.aspx?pagename=*Diospyros*_LT&projectid=17

[B44] Mangold J (2013) Plant identification basics. Montana State University Extension Mount Guide 9(13): 1–8. https://w.circlehgrowers.com/pdf/general/Plant-ID-Basics.pdf

[B45] Martellos S (2010) Multi-authored interactive identification keys: The FRIDA (FRiendly IDentificAtion) package. Taxon 59(3): 922–929. 10.1002/tax.593020

[B46] Mason J Parker M Vary LB Lowry PP II Hassold S Ruta G (2016) Malagasy precious hardwoods: scientific and technical assessment to meet CITES objectives. The World Bank, Washington DC. https://cites.org/sites/default/files/eng/cop/17/InfDocs/E-CoP17-Inf-88.pdf

[B47] Mestre E Lowry PP II Meeprom N Rakouth HN Linan AG Rabarimanarivo M Puglisi C (2024) Taxonomic studies of *Diospyros* (Ebenaceae) from the Malagasy Region. X. *Diospyrosmadagascariensis*, a new combination for the Malagasy populations previously included in *D.ferrea*. Novon 32: 46–51. 10.3417/2024896

[B48] Norton GA Patterson DJ Schneider M (2000) LucID: A multimedia educational tool for identification and diagnostics. International Journal of Innovation in Science and Mathematics Education 4(1): 1–7. https://openjournals.library.sydney.edu.au/index.php/CAL/article/view/6141

[B49] Pepke E Bowyer J Bratkovich S Fernholz K Frank M Groot H Howe J (2015) Impacts of policies to eliminate illegal timber trade. Minneapolis, US Dovetail Partners Inc., 19.

[B50] Perrier de la Bâthie H (1952a) Révision des Ébénacées de Madagascar et des Comores. Mémoire de l’Institut Scientifique de Madagascar, Série B. Biologie Végétale 4: 93–154.

[B51] Perrier de la Bâthie H (1952b) Ébénacées. In Humbert H (Ed.) Flore de Madagascar et des Comores, 165, Muséum national d’Histoire naturelle, Paris, 1–129.

[B52] Phillipson PP Wilding N Crameri S Andriambololonera S Rakotonirina N Rabarimanarivo M Manjato N (2023) Large tree species of *Dalbergia* from Madagascar. Species that are known or suspected to produce wood of commercial value. http://legacy.tropicos.org/projectwebportal.aspx?pagename=Dalbergia_LT&projectid=17

[B53] Pinel A Bouquin S Bourdon E Kerner A Vignes-Lebbe R (2017) Three years of Xper3 assessment: Towards sharing semantic taxonomic content of identification keys. Biodiversity Information Science and Standards. Biodiversity Information Science and Standards 1: e20382. 10.3897/tdwgproceedings.1.20382

[B54] POWO (2023) Plants of the World Online. Facilitated by the Royal Botanic Gardens, Kew. https://doi.org/plantsoftheworldonline.org

[B55] Puglisi C Jimbo T Hagwood A (2022) Two new species of *Diospyros* (Ebenaceae) from New Guinea. Edinburgh Journal of Botany 79: 1–10. 10.24823/EJB.2022.1879

[B56] Rabarimanarivo MN Rakotonirina NH Phillipson PB Lowry PP II (2015) Révision du genre *Ivodea* Capuron (Rutaceae), endémique de Madagascar et des Îles Comores. Adansonia série 3: 63–102. 10.5252/a2015n1a6

[B57] Rakotovao G Rabevohitra AR De Chatelperron PC Guibal D Gérard J (2012) Atlas des bois de Madagascar. CIRAD Edition Quaes Versailles.

[B58] Rakouth HN Randrianaivo R Andrianarivelo SAF Karatra DA Nombajanahary MZ Andriamiadana S Andriamiarisoa RL Bernard R Razakamalala R Andriambololonera S Rakouth B Lowry PP II (2023) Taxonomic Studies of *Diospyros* (Ebenaceae) from the Malagasy Region. IX. Clarification of Species Limits Between *D.clusiifolia* and *D.fuscovelutina*, and Establishment of a New Combination Based on a Name Previously Placed in Synonymy, *Tetraclisbaronii*. Novon 31: 156–162. 10.3417/2023848

[B59] Randriamalala H Liu Z (2010) Rosewood of Madagascar: Between democracy and conservation. Madagascar Conservation and Development 5(1): 11–22. 10.4314/mcd.v5i1.57336

[B60] Randriamalala H Rasarely E Ratsimbazafy J Brizzi A Ballet J Razakamanarina N Ratsifandrihamanana N Schuurman D (2011) Stocks de bois précieux de Madagascar quelle voie emprunter. Madagascar Conservation and Development 6(2): 88–96. 10.4314/mcd.v6i2.8

[B61] Ratsimbazafy C Newton DJ Ringuet S (2016) L’ île aux bois: le commerce de bois de rose et de bois d’ébène de Madagascar. TRAFFIC, Cambridge, 146. https://policycommons.net/artifacts/1935571/lile-aux-bois/2687341/

[B62] Schatz GE (2000) Endemism in the Malagasy tree flora. In: Lourenço WR Goodman SM (Eds) Diversité et Endémisme à Madagascar.Mémoire de la Société de Biogéographie,Paris, France, 1–9.

[B63] Schatz GE Lowry PP II (2018) Taxonomic studies of *Diospyros* (Ebenaceae) from the Malagasy region III. New species from the island of Nosy Mangabe in the Bay of Antongil. Novon 26(3): 272–286. 10.3417/2018209

[B64] Schatz GE Lowry PP II (2020) Taxonomic studies of *Diospyros* L. (Ebenaceae) from the Malagasy region IV Synoptic revision of the Squamosa group in Madagascar and the Comoro Islands. Adansonia sér 3(42): 201–218. 10.5252/adansonia2020v42a10

[B65] Schatz GE Lowry PP II Phillipson PB (2020) Taxonomic studies of *Diospyros* (Ebenaceae) from the Malagasy region V. Synoptic revision of the Bernieriana group in Madagascar and the Comoro Islands. Candollea 75(2): 203–218. 10.15553/c2020v752a5

[B66] Schatz GE Lowry PP II Rakouth HN (2021a) Taxonomic studies of *Diospyros* (Ebenaceae) from the Malagasy region. VIII. New species from the littoral forests of eastern Madagascar. Novon 29: 159–187. 10.3417/2021678

[B67] Schatz GE Lowry PP II Rakouth HN Randrianaivo R (2021b) Taxonomic studies of *Diospyros* (Ebenaceae) from the Malagasy region. VI. New species of large trees from Madagascar. Candollea 76(2): 201–236. 10.15553/c2021v762a3

[B68] Schuurman D Lowry PP II (2009) The Madagascar rosewood massacre. Madagascar Conservation and Development 4(2): 98–102. 10.4314/mcd.v4i2.48649

[B69] Stone CD (1996) La Convention de Rio de 1992 sur la diversité biologique. Le droit international face à l’éthique et à la politique de l’environnement. Georg, Genève, 119–133.

[B70] Tang D Zhang Q Xu L Guo D Luo Z (2019) Number of species and geographical distribution of *Diospyros* L. (Ebenaceae) in China. Horticultural Plant Journal 5(2): 59–69. 10.1016/j.hpj.2018.10.003

[B71] Ung V Dubus G Zaragüeta-Bagils RV-LR Vignes-Lebbe R (2010) Xper2: Introducing e-taxonomy. Bioinformatics 26(5): 703–704. 10.1093/bioinformatics/btp715 20053842

[B72] Vences M Wollenberg KC Vieites DR Lees DC (2009) Madagascar as a model region of species diversification. Trends in Ecology & Evolution 24(8): 456–465. 10.1016/j.tree.2009.03.011 19500874

[B73] Vignes-Lebbe R Chesselet P Diep Thi MH (2016) Xper3: new tools for collaborating, training, and transmitting knowledge on botanical phenotypes. In: Rakotoarisoa N, Blackmore S, Riera B (Eds) Proceedings of the UNESCO International Conference, Botanists of the twenty-first century: roles, challenges and opportunities, 228–239.

[B74] Waeber PO Wilmé L Ramamonjisoa B Garcia C Rakotomalala D Rabemananjara ZH Kull CA Ganzhorn JU Sorg J-P (2015) Dry forests in Madagascar: Neglected and under pressure. International Forestry Review 17(2): 127–148. 10.1505/146554815815834822

[B75] Wati RK van Vugt RR Gravendeel B (2018) A Linnaeus NG interactive key to the species of *Glomera* (Orchidaceae, Coelogyninae) from Southeast Asia. PhytoKeys 110: 9–22. 10.3897/phytokeys.110.28435 PMC6215026 30402036

[B76] Wilmé L Schuurman D Lowry PP II Raven PH (2009) Precious trees pay off – but who pays? An update. Document prepared for the COP15, Copenhagen Meeting, 7–15 December 2009. 10.4314/mcd.v5i1.57335

[B77] Wu F Gazo R Benes B Haviarova E (2021) Deep BarkID: A portable tree bark identification system by knowledge distillation. European Journal of Forest Research 140(6): 1391–1399. 10.1007/s10342-021-01407-7

[B78] Zuquim G Tuomisto H Prado J (2017) A free-access online key to identify Amazonian ferns. PhytoKeys 78: 1–15. 10.3897/phytokeys.78.11370 PMC5543271 28781548

